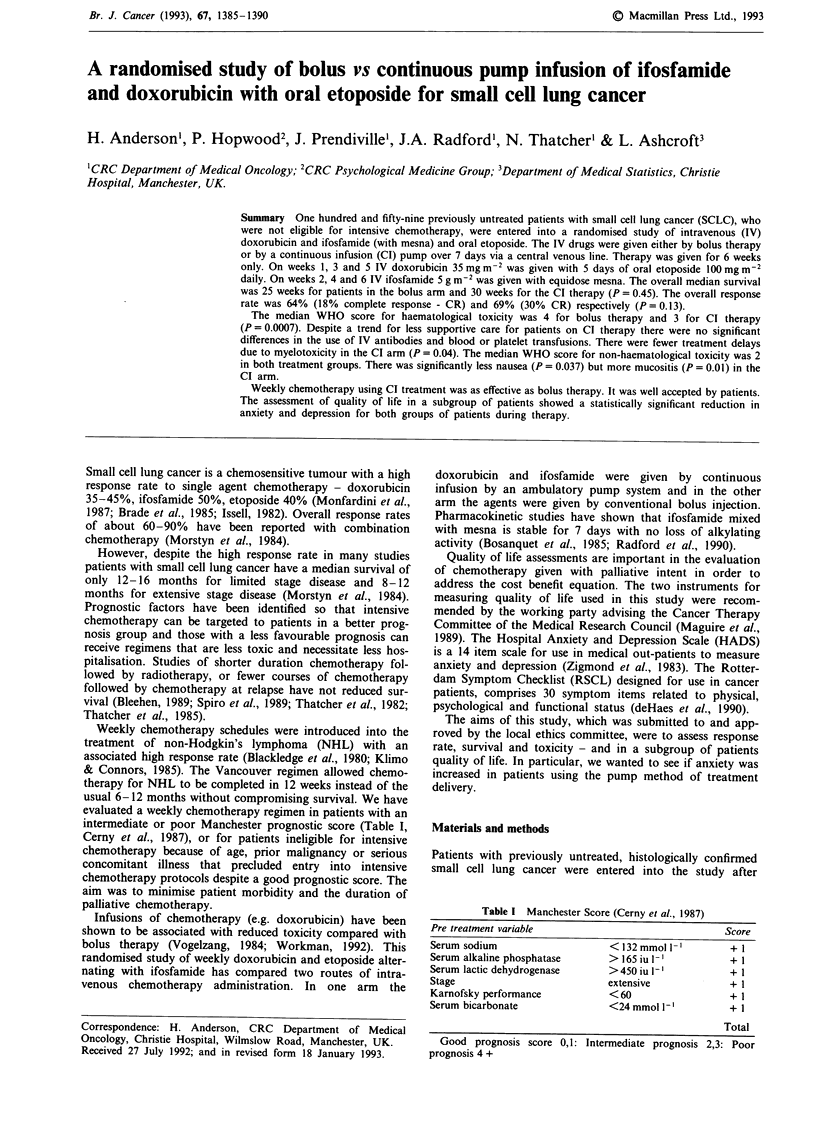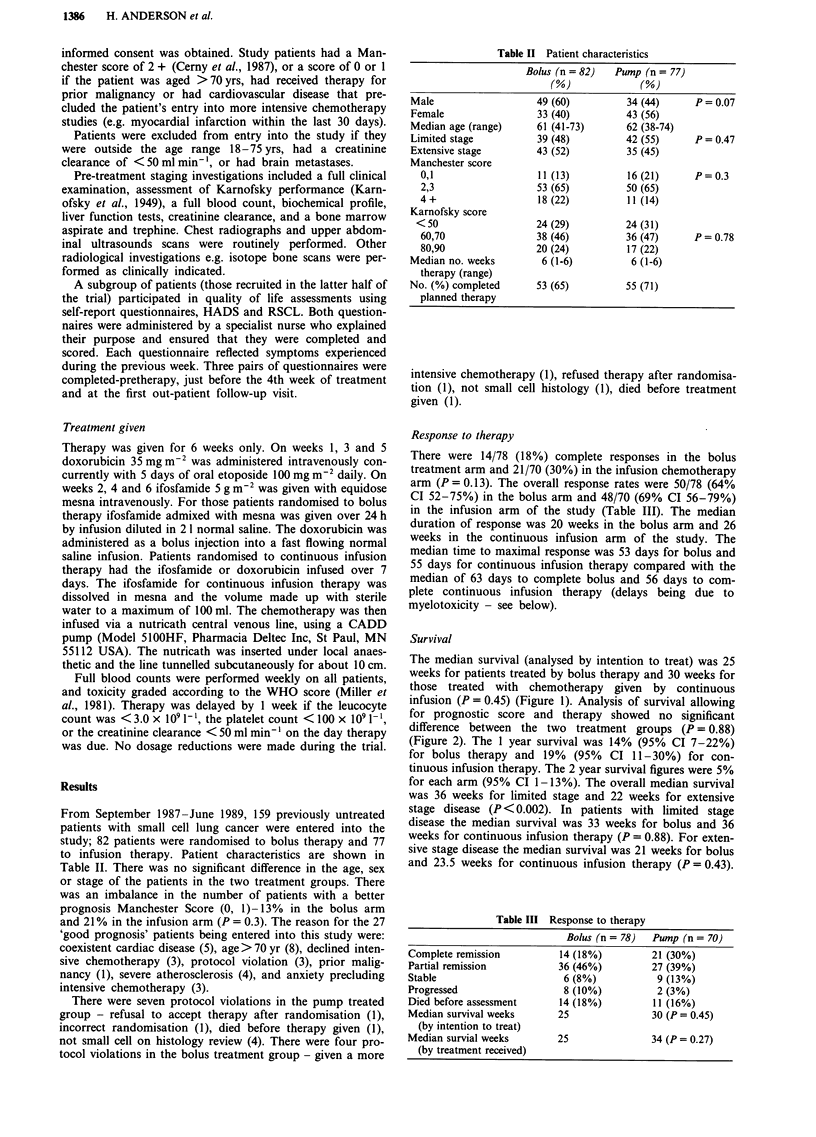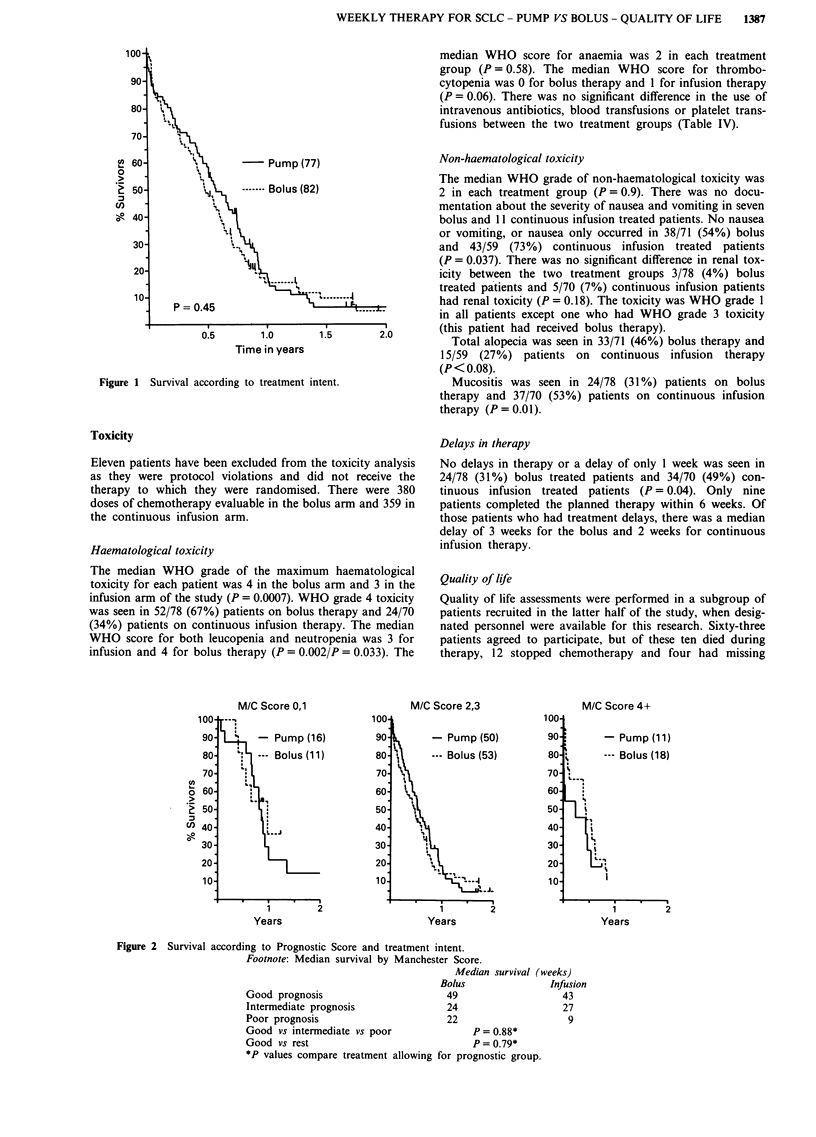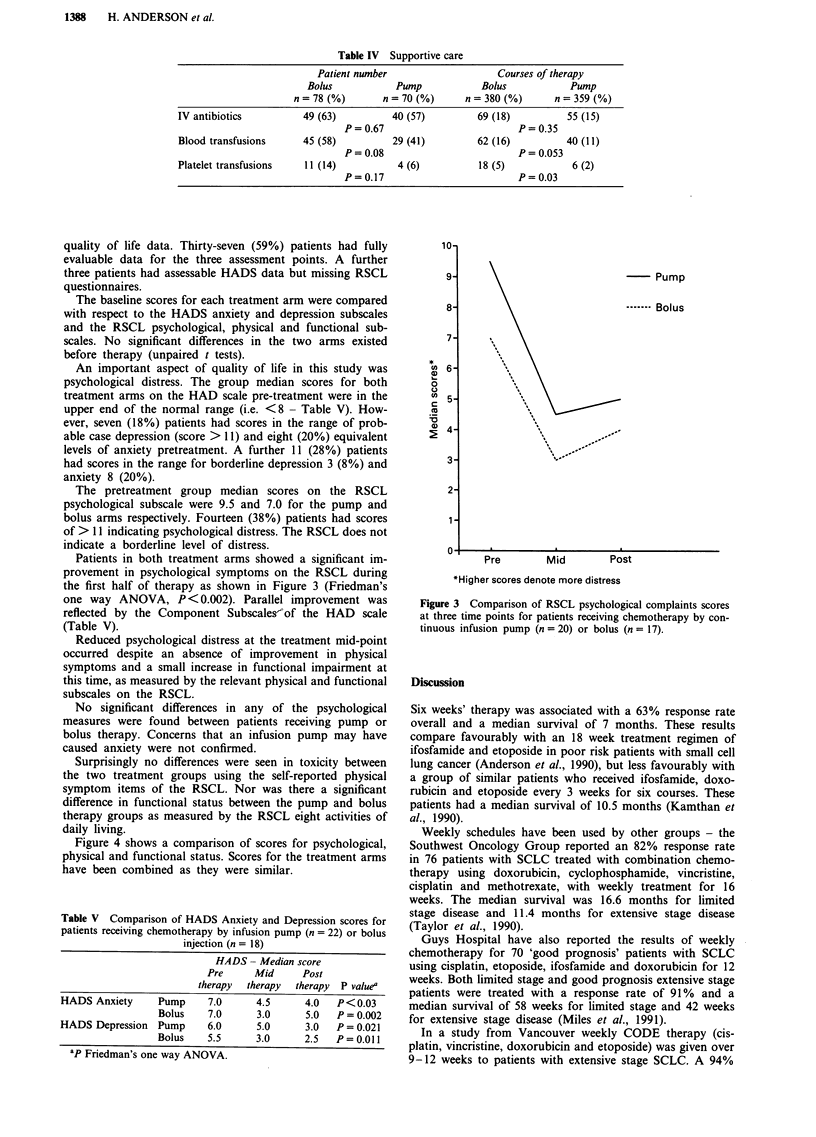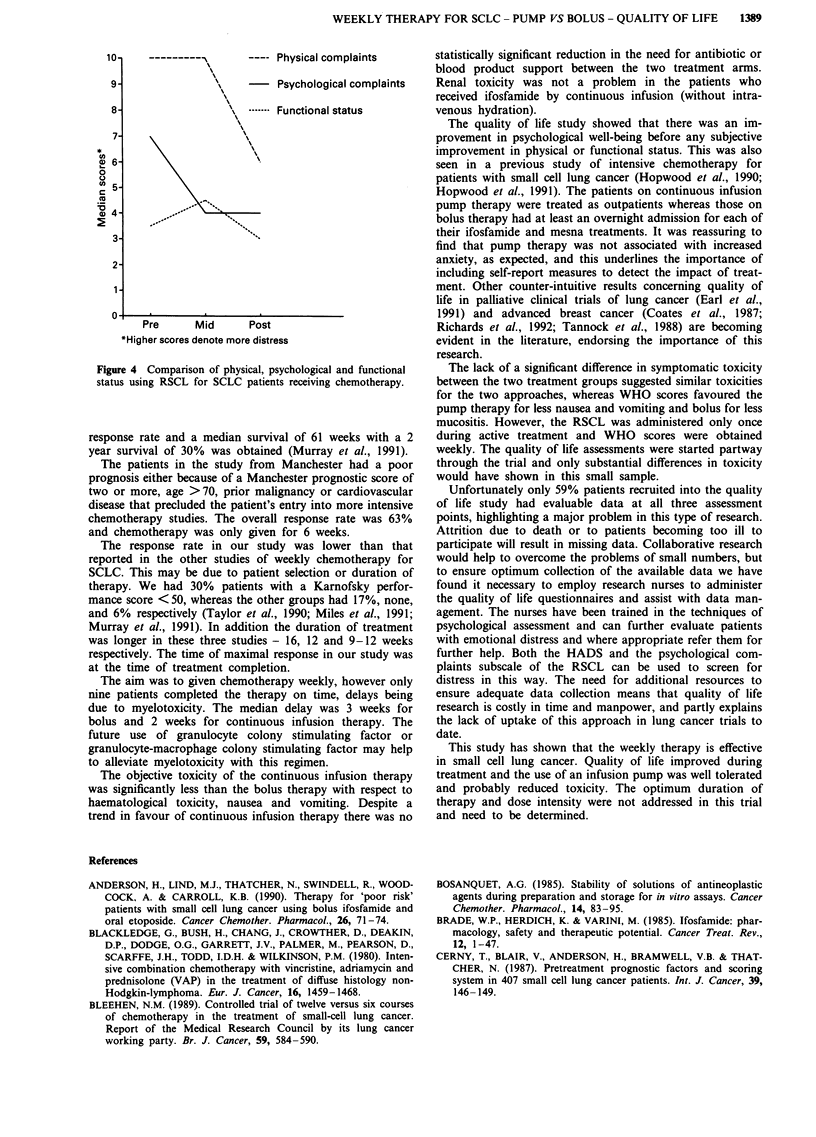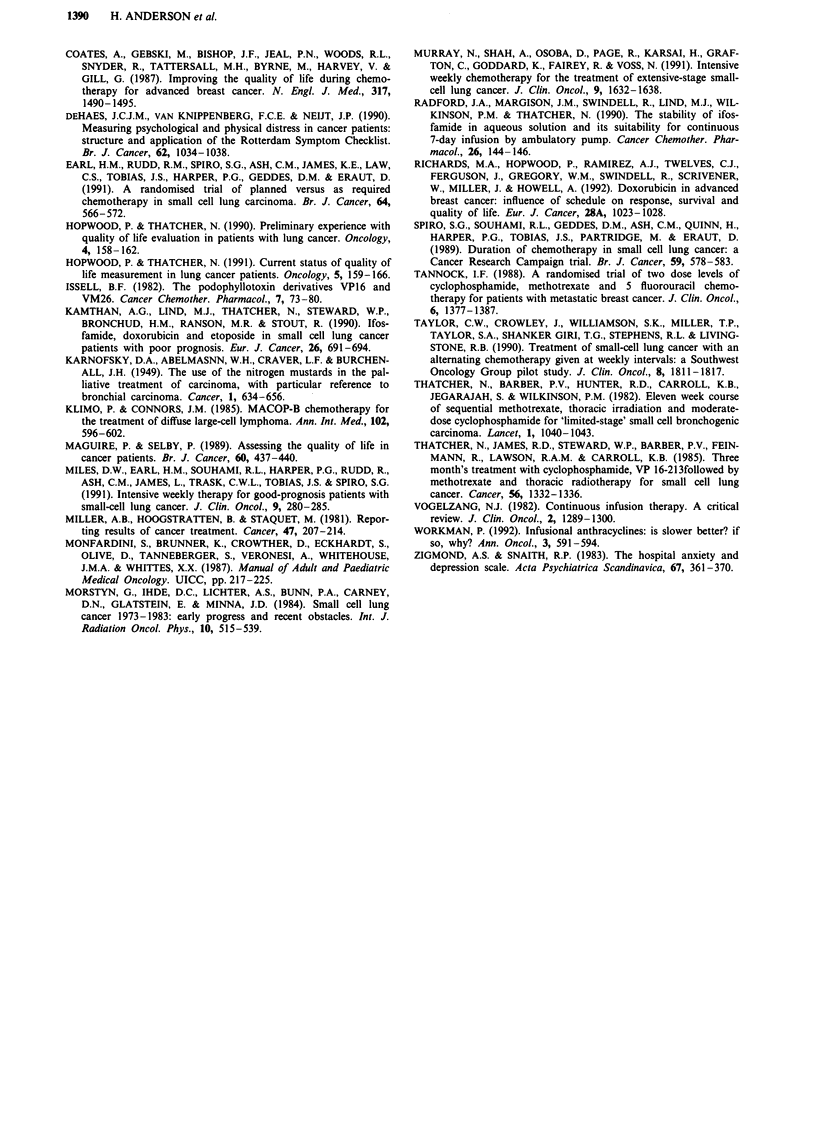# A randomised study of bolus vs continuous pump infusion of ifosfamide and doxorubicin with oral etoposide for small cell lung cancer.

**DOI:** 10.1038/bjc.1993.256

**Published:** 1993-06

**Authors:** H. Anderson, P. Hopwood, J. Prendiville, J. A. Radford, N. Thatcher, L. Ashcroft

**Affiliations:** CRC Department of Medical Oncology, Christie Hospital, Manchester, UK.

## Abstract

One hundred and fifty-nine previously untreated patients with small cell lung cancer (SCLC), who were not eligible for intensive chemotherapy, were entered into a randomised study of intravenous (i.v.) doxorubicin and ifosfamide (with mesna) and oral etoposide. The i.v. drugs were given either by bolus therapy or by a continuous infusion (CI) pump over 7 days via a central venous line. Therapy was given for 6 weeks only. On weeks 1, 3 and 5 IV doxorubicin 35 mg m-2 was given with 5 days of oral etoposide 100 mg m-2 daily. On weeks 2, 4 and 6 IV ifosfamide 5 g m-2 was given with equidose mesna. The overall median survival was 25 weeks for patients in the bolus arm and 30 weeks for the CI therapy (P = 0.45). The overall response rate was 64% (18% complete response-CR) and 69% (30% CR) respectively (P = 0.13). The median WHO score for haematological toxicity was 4 for bolus therapy and 3 for CI therapy (P = 0.0007). Despite a trend for less supportive care for patients on CI therapy there were no significant differences in the use of i.v. antibodies and blood or platelet transfusions. There were fewer treatment delays due to myelotoxicity in the CI arm (P = 0.04). The median WHO score for non-haematological toxicity was 2 in both treatment groups. There was significantly less nausea (P = 0.037) but more mucositis (P = 0.01) in the CI arm. Weekly chemotherapy using CI treatment was as effective as bolus therapy. It was well accepted by patients. The assessment of quality of life in a subgroup of patients showed a statistically significant reduction in anxiety and depression for both groups of patients during therapy.


Br. J. Cancer (1993), 67, 1385–1390 © Macmillan Press Ltd., 1993

### A randomised study of bolus *vs* continuous pump infusion of ifosfamide and doxorubicin with oral etoposide for small cell lung cancer

H. Anderson[1], P. Hopwood[2], J. Prendiville[1], J.A. Radford[1], N. Thatcher[1] & L. Ashcroft[3]

[1]*CRC Department of Medical Oncology;* [2]*CRC Psychological Medicine Group;* [3]*Department of Medical Statistics, Christie Hospital, Manchester, UK.*

**Summary** One hundred and fifty-nine previously untreated patients with small cell lung cancer (SCLC), who were not eligible for intensive chemotherapy, were entered into a randomised study of intravenous (IV) doxorubicin and ifosfamide (with mesna) and oral etoposide. The IV drugs were given either by bolus therapy or by a continuous infusion (CI) pump over 7 days via a central venous line. Therapy was given for 6 weeks only. On weeks 1, 3 and 5 IV doxorubicin 35 mg m$^{-2}$ was given with 5 days of oral etoposide 100 mg m$^{-2}$ daily. On weeks 2, 4 and 6 IV ifosfamide 5 g m$^{-2}$ was given with equidose mesna. The overall median survival was 25 weeks for patients in the bolus arm and 30 weeks for the CI therapy ($P = 0.45$). The overall response rate was 64% (18% complete response - CR) and 69% (30% CR) respectively ($P = 0.13$).

The median WHO score for haematological toxicity was 4 for bolus therapy and 3 for CI therapy ($P = 0.0007$). Despite a trend for less supportive care for patients on CI therapy there were no significant differences in the use of IV antibodies and blood or platelet transfusions. There were fewer treatment delays due to myelotoxicity in the CI arm ($P = 0.04$). The median WHO score for non-haematological toxicity was 2 in both treatment groups. There was significantly less nausea ($P = 0.037$) but more mucositis ($P = 0.01$) in the CI arm.

Weekly chemotherapy using CI treatment was as effective as bolus therapy. It was well accepted by patients. The assessment of quality of life in a subgroup of patients showed a statistically significant reduction in anxiety and depression for both groups of patients during therapy.

Small cell lung cancer is a chemosensitive tumour with a high response rate to single agent chemotherapy – doxorubicin 35–45%, ifosfamide 50%, etoposide 40% (Monfardini *et al.*, 1987; Brade *et al.*, 1985; Issell, 1982). Overall response rates of about 60–90% have been reported with combination chemotherapy (Morstyn *et al.*, 1984).

However, despite the high response rate in many studies patients with small cell lung cancer have a median survival of only 12–16 months for limited stage disease and 8–12 months for extensive stage disease (Morstyn *et al.*, 1984). Prognostic factors have been identified so that intensive chemotherapy can be targeted to patients in a better prognosis group and those with a less favourable prognosis can receive regimens that are less toxic and necessitate less hospitalisation. Studies of shorter duration chemotherapy followed by radiotherapy, or fewer courses of chemotherapy followed by chemotherapy at relapse have not reduced survival (Bleehen, 1989; Spiro *et al.*, 1989; Thatcher *et al.*, 1982; Thatcher *et al.*, 1985).

Weekly chemotherapy schedules were introduced into the treatment of non-Hodgkin's lymphoma (NHL) with an associated high response rate (Blackledge *et al.*, 1980; Klimo & Connors, 1985). The Vancouver regimen allowed chemotherapy for NHL to be completed in 12 weeks instead of the usual 6–12 months without compromising survival. We have evaluated a weekly chemotherapy regimen in patients with an intermediate or poor Manchester prognostic score (Table I, Cerny *et al.*, 1987), or for patients ineligible for intensive chemotherapy because of age, prior malignancy or serious concomitant illness that precluded entry into intensive chemotherapy protocols despite a good prognostic score. The aim was to minimise patient morbidity and the duration of palliative chemotherapy.

Infusions of chemotherapy (e.g. doxorubicin) have been shown to be associated with reduced toxicity compared with bolus therapy (Vogelzang, 1984; Workman, 1992). This randomised study of weekly doxorubicin and etoposide alternating with ifosfamide has compared two routes of intravenous chemotherapy administration. In one arm the doxorubicin and ifosfamide were given by continuous infusion by an ambulatory pump system and in the other arm the agents were given by conventional bolus injection. Pharmacokinetic studies have shown that ifosfamide mixed with mesna is stable for 7 days with no loss of alkylating activity (Bosanquet *et al.*, 1985; Radford *et al.*, 1990).

Quality of life assessments are important in the evaluation of chemotherapy given with palliative intent in order to address the cost benefit equation. The two instruments for measuring quality of life used in this study were recommended by the working party advising the Cancer Therapy Committee of the Medical Research Council (Maguire *et al.*, 1989). The Hospital Anxiety and Depression Scale (HADS) is a 14 item scale for use in medical out-patients to measure anxiety and depression (Zigmond *et al.*, 1983). The Rotterdam Symptom Checklist (RSCL) designed for use in cancer patients, comprises 30 symptom items related to physical, psychological and functional status (deHaes *et al.*, 1990).

The aims of this study, which was submitted to and approved by the local ethics committee, were to assess response rate, survival and toxicity – and in a subgroup of patients quality of life. In particular, we wanted to see if anxiety was increased in patients using the pump method of treatment delivery.

### Materials and methods

Patients with previously untreated, histologically confirmed small cell lung cancer were entered into the study after

**Table I** Manchester Score (Cerny *et al.*, 1987)

| *Pre treatment variable* | | *Score* |
|---|---|---|
| Serum sodium | $<$132 mmol l$^{-1}$ | +1 |
| Serum alkaline phosphatase | $>$165 iu l$^{-1}$ | +1 |
| Serum lactic dehydrogenase | $>$450 iu l$^{-1}$ | +1 |
| Stage | extensive | +1 |
| Karnofsky performance | $<$60 | +1 |
| Serum bicarbonate | $<$24 mmol l$^{-1}$ | +1 |
| | | Total |

Good prognosis score 0,1: Intermediate prognosis 2,3: Poor prognosis 4+

Correspondence: H. Anderson, CRC Department of Medical Oncology, Christie Hospital, Wilmslow Road, Manchester, UK.
Received 27 July 1992; and in revised form 18 January 1993.

informed consent was obtained. Study patients had a Manchester score of 2 + (Cerny et al., 1987), or a score of 0 or 1 if the patient was aged > 70 yrs, had received therapy for prior malignancy or had cardiovascular disease that precluded the patient's entry into more intensive chemotherapy studies (e.g. myocardial infarction within the last 30 days).

Patients were excluded from entry into the study if they were outside the age range 18–75 yrs, had a creatinine clearance of < 50 ml $min^{-1}$, or had brain metastases.

Pre-treatment staging investigations included a full clinical examination, assessment of Karnofsky performance (Karnofsky et al., 1949), a full blood count, biochemical profile, liver function tests, creatinine clearance, and a bone marrow aspirate and trephine. Chest radiographs and upper abdominal ultrasounds scans were routinely performed. Other radiological investigations e.g. isotope bone scans were performed as clinically indicated.

A subgroup of patients (those recruited in the latter half of the trial) participated in quality of life assessments using self-report questionnaires, HADS and RSCL. Both questionnaires were administered by a specialist nurse who explained their purpose and ensured that they were completed and scored. Each questionnaire reflected symptoms experienced during the previous week. Three pairs of questionnaires were completed-pretherapy, just before the 4th week of treatment and at the first out-patient follow-up visit.

*Treatment given*

Therapy was given for 6 weeks only. On weeks 1, 3 and 5 doxorubicin 35 mg $m^{-2}$ was administered intravenously concurrently with 5 days of oral etoposide 100 mg $m^{-2}$ daily. On weeks 2, 4 and 6 ifosfamide 5 g $m^{-2}$ was given with equidose mesna intravenously. For those patients randomised to bolus therapy ifosfamide admixed with mesna was given over 24 h by infusion diluted in 2 l normal saline. The doxorubicin was administered as a bolus injection into a fast flowing normal saline infusion. Patients randomised to continuous infusion therapy had the ifosfamide or doxorubicin infused over 7 days. The ifosfamide for continuous infusion therapy was dissolved in mesna and the volume made up with sterile water to a maximum of 100 ml. The chemotherapy was then infused via a nutricath central venous line, using a CADD pump (Model 5100HF, Pharmacia Deltec Inc, St Paul, MN 55112 USA). The nutricath was inserted under local anaesthetic and the line tunnelled subcutaneously for about 10 cm.

Full blood counts were performed weekly on all patients, and toxicity graded according to the WHO score (Miller et al., 1981). Therapy was delayed by 1 week if the leucocyte count was $< 3.0 \times 10^9 l^{-1}$, the platelet count $< 100 \times 10^9 l^{-1}$, or the creatinine clearance < 50 ml $min^{-1}$ on the day therapy was due. No dosage reductions were made during the trial.

### Results

From September 1987–June 1989, 159 previously untreated patients with small cell lung cancer were entered into the study; 82 patients were randomised to bolus therapy and 77 to infusion therapy. Patient characteristics are shown in Table II. There was no significant difference in the age, sex or stage of the patients in the two treatment groups. There was an imbalance in the number of patients with a better prognosis Manchester Score (0, 1)–13% in the bolus arm and 21% in the infusion arm ($P = 0.3$). The reason for the 27 'good prognosis' patients being entered into this study were: coexistent cardiac disease (5), age > 70 yr (8), declined intensive chemotherapy (3), protocol violation (3), prior malignancy (1), severe atherosclerosis (4), and anxiety precluding intensive chemotherapy (3).

There were seven protocol violations in the pump treated group – refusal to accept therapy after randomisation (1), incorrect randomisation (1), died before therapy given (1), not small cell on histology review (4). There were four protocol violations in the bolus treatment group – given a more intensive chemotherapy (1), refused therapy after randomisation (1), not small cell histology (1), died before treatment given (1).

**Table II** Patient characteristics

| | *Bolus* (n = *82*) (*%*) | *Pump* (n = *77*) (*%*) | |
|---|---|---|---|
| Male | 49 (60) | 34 (44) | $P = 0.07$ |
| Female | 33 (40) | 43 (56) | |
| Median age (range) | 61 (41-73) | 62 (38-74) | |
| Limited stage | 39 (48) | 42 (55) | $P = 0.47$ |
| Extensive stage | 43 (52) | 35 (45) | |
| Manchester score | | | |
| 0,1 | 11 (13) | 16 (21) | $P = 0.3$ |
| 2,3 | 53 (65) | 50 (65) | |
| 4 + | 18 (22) | 11 (14) | |
| Karnofsky score | | | |
| < 50 | 24 (29) | 24 (31) | |
| 60,70 | 38 (46) | 36 (47) | $P = 0.78$ |
| 80,90 | 20 (24) | 17 (22) | |
| Median no. weeks therapy (range) | 6 (1-6) | 6 (1-6) | |
| No. (%) completed planned therapy | 53 (65) | 55 (71) | |

*Response to therapy*

There were 14/78 (18%) complete responses in the bolus treatment arm and 21/70 (30%) in the infusion chemotherapy arm ($P = 0.13$). The overall response rates were 50/78 (64% CI 52–75%) in the bolus arm and 48/70 (69% CI 56–79%) in the infusion arm of the study (Table III). The median duration of response was 20 weeks in the bolus arm and 26 weeks in the continuous infusion arm of the study. The median time to maximal response was 53 days for bolus and 55 days for continuous infusion therapy compared with the median of 63 days to complete bolus and 56 days to complete continuous infusion therapy (delays being due to myelotoxicity – see below).

*Survival*

The median survival (analysed by intention to treat) was 25 weeks for patients treated by bolus therapy and 30 weeks for those treated with chemotherapy given by continuous infusion ($P = 0.45$) (Figure 1). Analysis of survival allowing for prognostic score and therapy showed no significant difference between the two treatment groups ($P = 0.88$) (Figure 2). The 1 year survival was 14% (95% CI 7–22%) for bolus therapy and 19% (95% CI 11–30%) for continuous infusion therapy. The 2 year survival figures were 5% for each arm (95% CI 1–13%). The overall median survival was 36 weeks for limited stage and 22 weeks for extensive stage disease ($P < 0.002$). In patients with limited stage disease the median survival was 33 weeks for bolus and 36 weeks for continuous infusion therapy ($P = 0.88$). For extensive stage disease the median survival was 21 weeks for bolus and 23.5 weeks for continuous infusion therapy ($P = 0.43$).

**Table III** Response to therapy

| | *Bolus* (n = *78*) | *Pump* (n = *70*) |
|---|---|---|
| Complete remission | 14 (18%) | 21 (30%) |
| Partial remission | 36 (46%) | 27 (39%) |
| Stable | 6 (8%) | 9 (13%) |
| Progressed | 8 (10%) | 2 (3%) |
| Died before assessment | 14 (18%) | 11 (16%) |
| Median survival weeks (by intention to treat) | 25 | 30 ($P = 0.45$) |
| Median survial weeks (by treatment received) | 25 | 34 ($P = 0.27$) |

**Figure 1** Survival according to treatment intent.

### Toxicity

Eleven patients have been excluded from the toxicity analysis as they were protocol violations and did not receive the therapy to which they were randomised. There were 380 doses of chemotherapy evaluable in the bolus arm and 359 in the continuous infusion arm.

### *Haematological toxicity*

The median WHO grade of the maximum haematological toxicity for each patient was 4 in the bolus arm and 3 in the infusion arm of the study ($P = 0.0007$). WHO grade 4 toxicity was seen in 52/78 (67%) patients on bolus therapy and 24/70 (34%) patients on continuous infusion therapy. The median WHO score for both leucopenia and neutropenia was 3 for infusion and 4 for bolus therapy ($P = 0.002/P = 0.033$). The median WHO score for anaemia was 2 in each treatment group ($P = 0.58$). The median WHO score for thrombocytopenia was 0 for bolus therapy and 1 for infusion therapy ($P = 0.06$). There was no significant difference in the use of intravenous antibiotics, blood transfusions or platelet transfusions between the two treatment groups (Table IV).

### *Non-haematological toxicity*

The median WHO grade of non-haematological toxicity was 2 in each treatment group ($P = 0.9$). There was no documentation about the severity of nausea and vomiting in seven bolus and 11 continuous infusion treated patients. No nausea or vomiting, or nausea only occurred in 38/71 (54%) bolus and 43/59 (73%) continuous infusion treated patients ($P = 0.037$). There was no significant difference in renal toxicity between the two treatment groups 3/78 (4%) bolus treated patients and 5/70 (7%) continuous infusion patients had renal toxicity ($P = 0.18$). The toxicity was WHO grade 1 in all patients except one who had WHO grade 3 toxicity (this patient had received bolus therapy).

Total alopecia was seen in 33/71 (46%) bolus therapy and 15/59 (27%) patients on continuous infusion therapy ($P < 0.08$).

Mucositis was seen in 24/78 (31%) patients on bolus therapy and 37/70 (53%) patients on continuous infusion therapy ($P = 0.01$).

### *Delays in therapy*

No delays in therapy or a delay of only 1 week was seen in 24/78 (31%) bolus treated patients and 34/70 (49%) continuous infusion treated patients ($P = 0.04$). Only nine patients completed the planned therapy within 6 weeks. Of those patients who had treatment delays, there was a median delay of 3 weeks for the bolus and 2 weeks for continuous infusion therapy.

### *Quality of life*

Quality of life assessments were performed in a subgroup of patients recruited in the latter half of the study, when designated personnel were available for this research. Sixty-three patients agreed to participate, but of these ten died during therapy, 12 stopped chemotherapy and four had missing

**Figure 2** Survival according to Prognostic Score and treatment intent.
*Footnote*: Median survival by Manchester Score.

| | *Median survival (weeks)* | |
|---|---|---|
| | *Bolus* | *Infusion* |
| Good prognosis | 49 | 43 |
| Intermediate prognosis | 24 | 27 |
| Poor prognosis | 22 | 9 |
| Good *vs* intermediate *vs* poor | $P = 0.88$* | |
| Good *vs* rest | $P = 0.79$* | |

**P* values compare treatment allowing for prognostic group.

**Table IV** Supportive care

| | Patient number | | | Courses of therapy | | |
|---|---|---|---|---|---|---|
| | Bolus n = 78 (%) | | Pump n = 70 (%) | Bolus n = 380 (%) | | Pump n = 359 (%) |
| IV antibiotics | 49 (63) | | 40 (57) | 69 (18) | | 55 (15) |
| | | P = 0.67 | | | P = 0.35 | |
| Blood transfusions | 45 (58) | | 29 (41) | 62 (16) | | 40 (11) |
| | | P = 0.08 | | | P = 0.053 | |
| Platelet transfusions | 11 (14) | | 4 (6) | 18 (5) | | 6 (2) |
| | | P = 0.17 | | | P = 0.03 | |

quality of life data. Thirty-seven (59%) patients had fully evaluable data for the three assessment points. A further three patients had assessable HADS data but missing RSCL questionnaires.

The baseline scores for each treatment arm were compared with respect to the HADS anxiety and depression subscales and the RSCL psychological, physical and functional subscales. No significant differences in the two arms existed before therapy (unpaired $t$ tests).

An important aspect of quality of life in this study was psychological distress. The group median scores for both treatment arms on the HAD scale pre-treatment were in the upper end of the normal range (i.e. $<8$ – Table V). However, seven (18%) patients had scores in the range of probable case depression (score $>11$) and eight (20%) equivalent levels of anxiety pretreatment. A further 11 (28%) patients had scores in the range for borderline depression 3 (8%) and anxiety 8 (20%).

The pretreatment group median scores on the RSCL psychological subscale were 9.5 and 7.0 for the pump and bolus arms respectively. Fourteen (38%) patients had scores of $>11$ indicating psychological distress. The RSCL does not indicate a borderline level of distress.

Patients in both treatment arms showed a significant improvement in psychological symptoms on the RSCL during the first half of therapy as shown in Figure 3 (Friedman's one way ANOVA, $P<0.002$). Parallel improvement was reflected by the Component Subscales of the HAD scale (Table V).

Reduced psychological distress at the treatment mid-point occurred despite an absence of improvement in physical symptoms and a small increase in functional impairment at this time, as measured by the relevant physical and functional subscales on the RSCL.

No significant differences in any of the psychological measures were found between patients receiving pump or bolus therapy. Concerns that an infusion pump may have caused anxiety were not confirmed.

Surprisingly no differences were seen in toxicity between the two treatment groups using the self-reported physical symptom items of the RSCL. Nor was there a significant difference in functional status between the pump and bolus therapy groups as measured by the RSCL eight activities of daily living.

Figure 4 shows a comparison of scores for psychological, physical and functional status. Scores for the treatment arms have been combined as they were similar.

**Table V** Comparison of HADS Anxiety and Depression scores for patients receiving chemotherapy by infusion pump (n = 22) or bolus injection (n = 18)

| | | HADS – Median score Pre therapy | Mid therapy | Post therapy | P value[a] |
|---|---|---|---|---|---|
| HADS Anxiety | Pump | 7.0 | 4.5 | 4.0 | $P<0.03$ |
| | Bolus | 7.0 | 3.0 | 5.0 | $P=0.002$ |
| HADS Depression | Pump | 6.0 | 5.0 | 3.0 | $P=0.021$ |
| | Bolus | 5.5 | 3.0 | 2.5 | $P=0.011$ |

[a]P Friedman's one way ANOVA.

**Figure 3** Comparison of RSCL psychological complaints scores at three time points for patients receiving chemotherapy by continuous infusion pump (n = 20) or bolus (n = 17).

### Discussion

Six weeks' therapy was associated with a 63% response rate overall and a median survival of 7 months. These results compare favourably with an 18 week treatment regimen of ifosfamide and etoposide in poor risk patients with small cell lung cancer (Anderson et al., 1990), but less favourably with a group of similar patients who received ifosfamide, doxorubicin and etoposide every 3 weeks for six courses. These patients had a median survival of 10.5 months (Kamthan et al., 1990).

Weekly schedules have been used by other groups – the Southwest Oncology Group reported an 82% response rate in 76 patients with SCLC treated with combination chemotherapy using doxorubicin, cyclophosphamide, vincristine, cisplatin and methotrexate, with weekly treatment for 16 weeks. The median survival was 16.6 months for limited stage disease and 11.4 months for extensive stage disease (Taylor et al., 1990).

Guys Hospital have also reported the results of weekly chemotherapy for 70 'good prognosis' patients with SCLC using cisplatin, etoposide, ifosfamide and doxorubicin for 12 weeks. Both limited stage and good prognosis extensive stage patients were treated with a response rate of 91% and a median survival of 58 weeks for limited stage and 42 weeks for extensive stage disease (Miles et al., 1991).

In a study from Vancouver weekly CODE therapy (cisplatin, vincristine, doxorubicin and etoposide) was given over 9–12 weeks to patients with extensive stage SCLC. A 94%

**Figure 4** Comparison of physical, psychological and functional status using RSCL for SCLC patients receiving chemotherapy.

response rate and a median survival of 61 weeks with a 2 year survival of 30% was obtained (Murray *et al.*, 1991).

The patients in the study from Manchester had a poor prognosis either because of a Manchester prognostic score of two or more, age >70, prior malignancy or cardiovascular disease that precluded the patient's entry into more intensive chemotherapy studies. The overall response rate was 63% and chemotherapy was only given for 6 weeks.

The response rate in our study was lower than that reported in the other studies of weekly chemotherapy for SCLC. This may be due to patient selection or duration of therapy. We had 30% patients with a Karnofsky performance score <50, whereas the other groups had 17%, none, and 6% respectively (Taylor *et al.*, 1990; Miles *et al.*, 1991; Murray *et al.*, 1991). In addition the duration of treatment was longer in these three studies - 16, 12 and 9-12 weeks respectively. The time of maximal response in our study was at the time of treatment completion.

The aim was to given chemotherapy weekly, however only nine patients completed the therapy on time, delays being due to myelotoxicity. The median delay was 3 weeks for bolus and 2 weeks for continuous infusion therapy. The future use of granulocyte colony stimulating factor or granulocyte-macrophage colony stimulating factor may help to alleviate myelotoxicity with this regimen.

The objective toxicity of the continuous infusion therapy was significantly less than the bolus therapy with respect to haematological toxicity, nausea and vomiting. Despite a trend in favour of continuous infusion therapy there was no statistically significant reduction in the need for antibiotic or blood product support between the two treatment arms. Renal toxicity was not a problem in the patients who received ifosfamide by continuous infusion (without intravenous hydration).

The quality of life study showed that there was an improvement in psychological well-being before any subjective improvement in physical or functional status. This was also seen in a previous study of intensive chemotherapy for patients with small cell lung cancer (Hopwood *et al.*, 1990; Hopwood *et al.*, 1991). The patients on continuous infusion pump therapy were treated as outpatients whereas those on bolus therapy had at least an overnight admission for each of their ifosfamide and mesna treatments. It was reassuring to find that pump therapy was not associated with increased anxiety, as expected, and this underlines the importance of including self-report measures to detect the impact of treatment. Other counter-intuitive results concerning quality of life in palliative clinical trials of lung cancer (Earl *et al.*, 1991) and advanced breast cancer (Coates *et al.*, 1987; Richards *et al.*, 1992; Tannock *et al.*, 1988) are becoming evident in the literature, endorsing the importance of this research.

The lack of a significant difference in symptomatic toxicity between the two treatment groups suggested similar toxicities for the two approaches, whereas WHO scores favoured the pump therapy for less nausea and vomiting and bolus for less mucositis. However, the RSCL was administered only once during active treatment and WHO scores were obtained weekly. The quality of life assessments were started partway through the trial and only substantial differences in toxicity would have shown in this small sample.

Unfortunately only 59% patients recruited into the quality of life study had evaluable data at all three assessment points, highlighting a major problem in this type of research. Attrition due to death or to patients becoming too ill to participate will result in missing data. Collaborative research would help to overcome the problems of small numbers, but to ensure optimum collection of the available data we have found it necessary to employ research nurses to administer the quality of life questionnaires and assist with data management. The nurses have been trained in the techniques of psychological assessment and can further evaluate patients with emotional distress and where appropriate refer them for further help. Both the HADS and the psychological complaints subscale of the RSCL can be used to screen for distress in this way. The need for additional resources to ensure adequate data collection means that quality of life research is costly in time and manpower, and partly explains the lack of uptake of this approach in lung cancer trials to date.

This study has shown that the weekly therapy is effective in small cell lung cancer. Quality of life improved during treatment and the use of an infusion pump was well tolerated and probably reduced toxicity. The optimum duration of therapy and dose intensity were not addressed in this trial and need to be determined.

### References

ANDERSON, H., LIND, M.J., THATCHER, N., SWINDELL, R., WOODCOCK, A. & CARROLL, K.B. (1990). Therapy for 'poor risk' patients with small cell lung cancer using bolus ifosfamide and oral etoposide. *Cancer Chemother. Pharmacol.*, **26**, 71-74.

BLACKLEDGE, G., BUSH, H., CHANG, J., CROWTHER, D., DEAKIN, D.P., DODGE, O.G., GARRETT, J.V., PALMER, M., PEARSON, D., SCARFFE, J.H., TODD, I.D.H. & WILKINSON, P.M. (1980). Intensive combination chemotherapy with vincristine, adriamycin and prednisolone (VAP) in the treatment of diffuse histology non-Hodgkin-lymphoma. *Eur. J. Cancer*, **16**, 1459-1468.

BLEEHEN, N.M. (1989). Controlled trial of twelve versus six courses of chemotherapy in the treatment of small-cell lung cancer. Report of the Medical Research Council by its lung cancer working party. *Br. J. Cancer*, **59**, 584-590.

BOSANQUET, A.G. (1985). Stability of solutions of antineoplastic agents during preparation and storage for *in vitro* assays. *Cancer Chemother. Pharmacol.*, **14**, 83-95.

BRADE, W.P., HERDICH, K. & VARINI, M. (1985). Ifosfamide: pharmacology, safety and therapeutic potential. *Cancer Treat. Rev.*, **12**, 1-47.

CERNY, T., BLAIR, V., ANDERSON, H., BRAMWELL, V.B. & THATCHER, N. (1987). Pretreatment prognostic factors and scoring system in 407 small cell lung cancer patients. *Int. J. Cancer*, **39**, 146-149.

COATES, A., GEBSKI, M., BISHOP, J.F., JEAL, P.N., WOODS, R.L., SNYDER, R., TATTERSALL, M.H., BYRNE, M., HARVEY, V. & GILL, G. (1987). Improving the quality of life during chemotherapy for advanced breast cancer. *N. Engl. J. Med.*, **317**, 1490–1495.

DEHAES, J.C.J.M., VAN KNIPPENBERG, F.C.E. & NEIJT, J.P. (1990). Measuring psychological and physical distress in cancer patients: structure and application of the Rotterdam Symptom Checklist. *Br. J. Cancer*, **62**, 1034–1038.

EARL, H.M., RUDD, R.M., SPIRO, S.G., ASH, C.M., JAMES, K.E., LAW, C.S., TOBIAS, J.S., HARPER, P.G., GEDDES, D.M. & ERAUT, D. (1991). A randomised trial of planned versus as required chemotherapy in small cell lung carcinoma. *Br. J. Cancer*, **64**, 566–572.

HOPWOOD, P. & THATCHER, N. (1990). Preliminary experience with quality of life evaluation in patients with lung cancer. *Oncology*, **4**, 158–162.

HOPWOOD, P. & THATCHER, N. (1991). Current status of quality of life measurement in lung cancer patients. *Oncology*, **5**, 159–166.

ISSELL, B.F. (1982). The podophyllotoxin derivatives VP16 and VM26. *Cancer Chemother. Pharmacol.*, **7**, 73–80.

KAMTHAN, A.G., LIND, M.J., THATCHER, N., STEWARD, W.P., BRONCHUD, H.M., RANSON, M.R. & STOUT, R. (1990). Ifosfamide, doxorubicin and etoposide in small cell lung cancer patients with poor prognosis. *Eur. J. Cancer*, **26**, 691–694.

KARNOFSKY, D.A., ABELMASNN, W.H., CRAVER, L.F. & BURCHENALL, J.H. (1949). The use of the nitrogen mustards in the palliative treatment of carcinoma, with particular reference to bronchial carcinoma. *Cancer*, **1**, 634–656.

KLIMO, P. & CONNORS, J.M. (1985). MACOP-B chemotherapy for the treatment of diffuse large-cell lymphoma. *Ann. Int. Med.*, **102**, 596–602.

MAGUIRE, P. & SELBY, P. (1989). Assessing the quality of life in cancer patients. *Br. J. Cancer*, **60**, 437–440.

MILES, D.W., EARL, H.M., SOUHAMI, R.L., HARPER, P.G., RUDD, R., ASH, C.M., JAMES, L., TRASK, C.W.L., TOBIAS, J.S. & SPIRO, S.G (1991). Intensive weekly therapy for good-prognosis patients with small-cell lung cancer. *J. Clin. Oncol.*, **9**, 280–285.

MILLER, A.B., HOOGSTRATTEN, B. & STAQUET, M. (1981). Reporting results of cancer treatment. *Cancer*, **47**, 207–214.

MONFARDINI, S., BRUNNER, K., CROWTHER, D., ECKHARDT, S., OLIVE, D., TANNEBERGER, S., VERONESI, A., WHITEHOUSE, J.M.A. & WHITTES, X.X. (1987). *Manual of Adult and Paediatric Medical Oncology*. UICC, pp. 217–225.

MORSTYN, G., IHDE, D.C., LICHTER, A.S., BUNN, P.A., CARNEY, D.N., GLATSTEIN, E. & MINNA, J.D. (1984). Small cell lung cancer 1973–1983: early progress and recent obstacles. *Int. J. Radiation Oncol. Phys.*, **10**, 515–539.

MURRAY, N., SHAH, A., OSOBA, D., PAGE, R., KARSAI, H., GRAFTON, C., GODDARD, K., FAIREY, R. & VOSS, N. (1991). Intensive weekly chemotherapy for the treatment of extensive-stage small-cell lung cancer. *J. Clin. Oncol.*, **9**, 1632–1638.

RADFORD, J.A., MARGISON, J.M., SWINDELL, R., LIND, M.J., WILKINSON, P.M. & THATCHER, N. (1990). The stability of ifosfamide in aqueous solution and its suitability for continuous 7-day infusion by ambulatory pump. *Cancer Chemother. Pharmacol.*, **26**, 144–146.

RICHARDS, M.A., HOPWOOD, P., RAMIREZ, A.J., TWELVES, C.J., FERGUSON, J., GREGORY, W.M., SWINDELL, R., SCRIVENER, W., MILLER, J. & HOWELL, A. (1992). Doxorubicin in advanced breast cancer: influence of schedule on response, survival and quality of life. *Eur. J. Cancer*, **28A**, 1023–1028.

SPIRO, S.G., SOUHAMI, R.L., GEDDES, D.M., ASH, C.M., QUINN, H., HARPER, P.G., TOBIAS, J.S., PARTRIDGE, M. & ERAUT, D. (1989). Duration of chemotherapy in small cell lung cancer: a Cancer Research Campaign trial. *Br. J. Cancer*, **59**, 578–583.

TANNOCK, I.F. (1988). A randomised trial of two dose levels of cyclophosphamide, methotrexate and 5 fluorouracil chemotherapy for patients with metastatic breast cancer. *J. Clin. Oncol.*, **6**, 1377–1387.

TAYLOR, C.W., CROWLEY, J., WILLIAMSON, S.K., MILLER, T.P., TAYLOR, S.A., SHANKER GIRI, T.G., STEPHENS, R.L. & LIVINGSTONE, R.B. (1990). Treatment of small-cell lung cancer with an alternating chemotherapy given at weekly intervals: a Southwest Oncology Group pilot study. *J. Clin. Oncol.*, **8**, 1811–1817.

THATCHER, N., BARBER, P.V., HUNTER, R.D., CARROLL, K.B., JEGARAJAH, S. & WILKINSON, P.M. (1982). Eleven week course of sequential methotrexate, thoracic irradiation and moderate-dose cyclophosphamide for 'limited-stage' small cell bronchogenic carcinoma. *Lancet*, **1**, 1040–1043.

THATCHER, N., JAMES, R.D., STEWARD, W.P., BARBER, P.V., FEINMANN, R., LAWSON, R.A.M. & CARROLL, K.B. (1985). Three month's treatment with cyclophosphamide, VP 16-213followed by methotrexate and thoracic radiotherapy for small cell lung cancer. *Cancer*, **56**, 1332–1336.

VOGELZANG, N.J. (1982). Continuous infusion therapy. A critical review. *J. Clin. Oncol.*, **2**, 1289–1300.

WORKMAN, P. (1992). Infusional anthracyclines: is slower better? if so, why? *Ann. Oncol.*, **3**, 591–594.

ZIGMOND, A.S. & SNAITH, R.P. (1983). The hospital anxiety and depression scale. *Acta Psychiatrica Scandinavica*, **67**, 361–370.